# Prevalence and Determinants of Diarrhea, Fever, and Coexistence of Diarrhea and Fever in Children Under-Five in Bangladesh

**DOI:** 10.3390/children10111829

**Published:** 2023-11-20

**Authors:** Md. Shariful Islam, Mohammad Rocky Khan Chowdhury, Farzana Akhter Bornee, Hasina Akhter Chowdhury, Baki Billah, Manzur Kader, Mamunur Rashid

**Affiliations:** 1 Department of Public Health, First Capital University of Bangladesh, Chuadanga 7200, Bangladesh; pmsi.sharif@fcub.edu.bd (M.S.I.); mohammad.chowdhury2@monash.edu (M.R.K.C.); 2 Department of Epidemiology and Preventive Medicine, School of Public Health and Preventive Medicine, Faculty of Medicine, Nursing and Health Sciences, Monash University, Melbourne 3004, Australia; hasina.chowdhury@monash.edu (H.A.C.); baki.billah@monash.edu (B.B.); 3 Department of Pediatrics, Bangabandhu Sheikh Mujib Medical University, Dhaka 1000, Bangladesh; bornee_dmc66@yahoo.com; 4 Department of Medicine, Clinical Epidemiology Division, Karolinska Institutet, Solna, 17177 Stockholm, Sweden; manzur.kader@outlook.com; 5 Department of Public Health and Sports Science, Faculty of Health and Occupational Studies, University of Gävle, Kungsbacksvägen 47, 80176 Gävle, Sweden

**Keywords:** children, morbidity, comorbidity, determinants, Bangladesh

## Abstract

Diarrhea and fever are prevalent childhood illnesses with potentially severe consequences, especially when they co-occur. This study investigates the prevalence and determinants of diarrhea, fever, and their coexistence among children under-five in Bangladesh. Data from the 2017–2018 Bangladesh Demography and Health Survey (BDHS) were analyzed using multivariable stepwise logistic regression with backward selection. This study found that 5.0% for diarrhea, 34.0% for fever, and 3.0% for the coexistence of both illnesses. Common factors associated with childhood diarrhea and fever included the child’s age (12–23 months), and the mother’s education. Diarrhea was associated with households with improved water sources and children in the Barisal division, while fever was linked to underweight children and those from more affluent backgrounds. The coexistence of both was significantly linked to underweight children, higher birth orders, and children from the Rajshahi division. Notably, child illnesses were associated with parental education, higher socio-economic status, and access to improved drinking water sources. Diarrhea affects one in 20 children, fever affects one in three, and the coexistence of both conditions affects one in 35 children in Bangladesh. The findings need further research and policy reviews to develop effective interventions and improve child health in Bangladesh.

## 1. Introduction

Diarrhea and fever in children represent significant public health challenges in low- and middle-income countries. Globally, diarrhea alone accounts for approximately 0.5 million deaths among children under the age of five annually and stands as the second most common cause of mortality in this age group [1,2]. Globally, there are nearly 1.7 billion cases of childhood diarrhea recorded every year [2,3]. According to UNICEF (2021), approximately 5 million under-five children died worldwide, of which, about 30% were attributed to infectious diseases, many of which present with diarrhea and fever [4,5]. Fever in children occurs mostly due to an infection (such as strep throat, the flu, chickenpox, pneumonia), inflammation and trauma [6], malaria, dengue, chikungunya, typhoid, and diarrhea [7]. Developing countries often face a high burden of childhood illness due to several factors, including poor living conditions, limited access to healthcare, inadequate nutrition, and exposure to infectious diseases [8]. Diarrhea and fever are common health problems among children under-five in Bangladesh, as in many other developing countries [9].

Both the fever and diarrhea are among the top three causes of mortality among children in Bangladesh [1,7]. Although the under-five child mortality has decreased from 44/1000 live births in 2011 to 27/1000 in 2021 [10,11] and achieved the Millennium Development Goal-4 (MDG-4) target of 48/1000 live births by the year 2015, childhood illness has not declined at the same pace [1]. In Bangladesh, each year, approximately half a million children die due to diarrhea, surpassing the combined mortality rates of AIDS, malaria, and measles [7]. However, metrics with regard to under-five child deaths due to fever are yet to be revealed [12].

Factors associated with childhood diarrhea and fever are multifaceted, and both outcomes had complex interplay with maternal-child factors (e.g., mothers’ education, unemployment status of mothers, age of children, infant and child feeding practice), environmental factors (e.g., drinking water, type of toilet facilities, household waste, and hygiene) and socioeconomic status in developing countries including Bangladesh, Ghana, Senegal, and Ethiopia [1,9,13,14,15,16]. However, in those studies, a preselection process was employed from the list of selected variables to adjust the models and identify the significant variables associated with childhood illness. In current study, stepwise logistic regression analysis with backward elimination was utilized, incorporating all selected variables in the models, to determine the optimal list of significant variables, which had not been thoroughly documented before. Furthermore, factors associated with the coexistence of diarrhea and fever among under-five children are still underreported in Bangladesh. A previous research conducted in Bangladesh found an association between the simultaneous presence of diarrhea and cough and factors such as low socio-economic status and untreated water supply [17]. Diarrhea and fever when they occur together in children can create an adverse health hazard, such as malnutrition, increased risk of infections, electrolyte imbalances, reduced cognitive function, and others [18,19,20]. To the best of our knowledge, there has been no study conducted in Bangladesh using the most recent nationally representative survey to explore the factors linked to the coexistence of diarrhea and fever. Therefore, this study aims to determine the factors associated with diarrhea, fever, and the coexistence of diarrhea and fever among under-five children in Bangladesh based on the most recent Bangladesh Demographic and Health Survey. This research will shed light on both typical and atypical factors related to the presence of diarrhea, fever, and their simultaneous occurrence. Such insights will contribute to the development of policies tailored to specific contexts.

## 2. Materials and Methods

### 2.1. Study Design and Participants

Cross-sectional data from a nationally representative sample namely the Bangladesh Demographic and Health Survey 2017–2018 (BDHS 2017–2018) was used in this study. In BDHS 2017–2018, the data collection was started in October 2017 and finished in March 2018.

**Figure 1:**
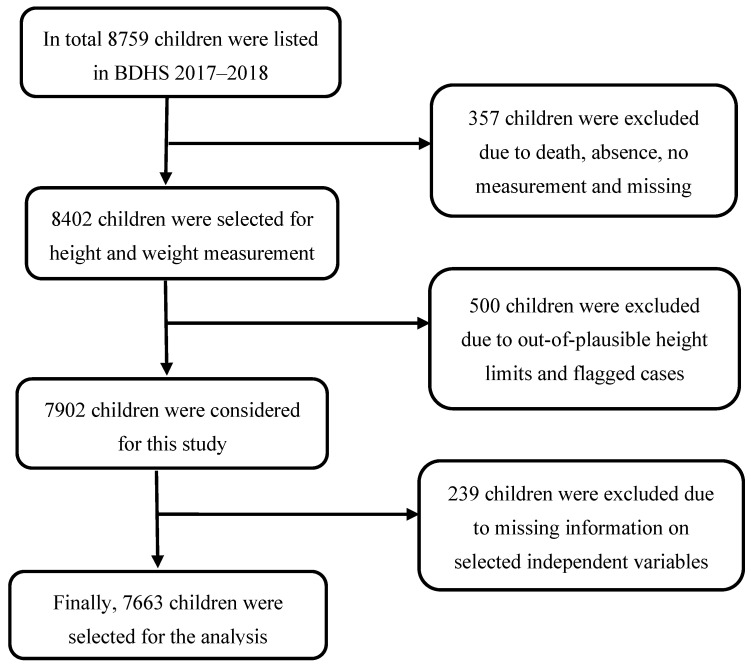
Selection of sample size.

In BDHS 2017–2018 [7], multistage stratified sampling technique was used to collect the data. At the first stage, the BDHS selected a total of 672 primary sampling units (PSUs) (192 PSUs from urban and 480 PSUs from rural) from 293,579 PSUs (according to the national census 2011) using probability proportional to enumeration area (EA) size. In the second stage, 20,160 households were selected for data collection with 30 households from each selected PSU using an equal probability systematic sampling technique. This multistage sampling technique, including its sampling weight, helps reduce potential sampling bias. In the BDHS data, sample weights were calculated in each sampling stage, and each cluster and stratum were considered that had been adjusted for non-response to obtain the final standard weights. In addition, all ever-married women aged 15–49 years with children aged less than 5 years from the preselected households were interviewed without replacement and change in the implementing stage to prevent selection bias. Women verbal consent were taken for collecting children’s data, including demography, health, and nutrition. A total of 8759 children under-five were listed, and a further 7663 children were selected for analysis after excluding missing information (Figure 1).

### 2.2. Outcome Variables

The outcome variables were diarrhea, fever, and the coexistence of diarrhea and fever among under-five children. Responses were coded 1 for experienced these illnesses (diarrhea, fever, and the coexistence of diarrhea and fever) and 0 for no illness. In BDHS 2017–2018, Diarrhea refers to children who had diarrhea in the 2 weeks preceding the survey and received oral rehydration solution (ORS), and advice or treatment from health facilities or qualified health providers [7]. Fever refers to children who had a fever prior to two weeks of the survey and received advice or treatment from health facilities or qualified health providers [7]. The coexistence of diarrhea and fever refers to the simultaneous presence of both illnesses in a child in the two weeks preceding the survey.

### 2.3. Independent Variables and Operational Definition

Variables found significantly associated with child illness in various studies were included in this study [1,9,13,14,15,16,17,18,19,21,22]. Child characteristics include the age of the children (0–11 months, 12–23 months, 24–35 months, 36–47 months, 48–59 months), sex of the children (male, female), underweight children (no, yes), birth order of children (first, second, third, fourth and above), and small birth weight (no, yes, not weighted). Variables including maternal characteristics were age of the mothers (in years) (15–19, 20–24, 25–29, 30–34, 34–39, ≥ 40), parents’ education (both parents uneducated, only father uneducated, only mother uneducated, both parents educated), mothers’ currently working (no, yes), mothers received antenatal care (no, yes), mothers received postnatal care (no, yes), mother’s decision-making autonomy (practiced, not practiced). Household characteristics include mass media exposure (no, yes), source of drinking water (improved, unimproved), type of toilet facility (improved, unimproved), and solid waste use in cooking (no, yes). Finally, contextual factors were wealth index (poorest, poorer, middle, richer, richest), place of residence (urban, rural), and region of residence (Barisal, Chattogram, Dhaka, Khulna, Mymensingh, Rajshahi, Rangpur, Sylhet). Supplementary Materials, Table S1 contains the operational definitions for reference.

### 2.4. Statistical Analyses

The background characteristics of the respondents were assessed using descriptive statistics. Bivariate analysis (Chi-square test) was used to explore the prevalence of diarrhea, fever, and the coexistence of diarrhea and fever. The stepwise logistic regression with backward elimination analysis was used to identify the factors associated with diarrhea, fever, and the coexistence of diarrhea and fever. Variables found significant at *p* < 0.25 in the Chi-square test were entered into the regression analysis [23,24,25]. The results of regression analysis were assessed using odds ratios (ORs) and 95% confidence interval (CI) where *p* < 0.05. To control the effect of the complex survey design (multistage sampling stage including its sampling weight), bivariate analysis in this study was performed using Stata’s ‘svyset’ command. Variance Inflation Factor (VIF) was used to evaluate the possible collinearity, and there was no multicollinearity problem identified among the study variables (VIF < 5 for all variables). All statistical analyses were performed in Stata version 17 (StataCorp LP, College Station, TX, USA).

## 3. Results

### 3.1. Background Characteristics

More than 40% of the children were in the age group of 0–23 months, and slightly more than half (52.2%) were male. A total of 35.0% of mothers fell within the age bracket of 20–24 years. The proportion of mothers with no formal education was just 7.0%, combining both 3.8% and 3.2%. Furthermore, more than 40.0% of children belonged to poor socio-economic status. Approximately two-thirds (66.0%) of children were living in rural areas of Bangladesh (Table 1).

### 3.2. Prevalence of Diarrhea, Fever, and Coexistence of Diarrhea and Fever

The overall prevalence of diarrhea, fever, and coexistence of diarrhea and fever among children under 5 were approximately 5.0%, 34.0%, and 3.0%, respectively. Children aged 12–23 months (9.1%), children of mothers with no formal education (when fathers were educated) (8.7%), children of teenaged (15–19 years) mothers (6.4%), and children from the socio-economically middle class (6.1%) had a significantly higher prevalence of diarrhea (Table 2).

Similarly, fever was more common among children aged 12–23 months with a prevalence of 40.0%, and those who were underweight (38.5%). It was also higher in children whose mothers received postnatal care (38.2%). Children of fourth and higher birth order had a significantly higher prevalence of fever (37.0%) (Table 2).

Children aged 12–23 months (4.8%) and those with a second birth order (3.4%) had a significantly higher prevalence of coexistence of fever and diarrhea (Table 2).

Children aged 12–23 months had a significantly higher likelihood of getting diarrhea with an odds of 6.78 (Adjusted odds ratio (AOR): 6.78, 95% CI: 4.35–10.54, *p* < 0.001) compared to children aged 48–59 months. Children of mothers with no formal education (AOR = 1.94, 95% CI = 1.23–3.07, *p* = 0.004) and children from the Barisal division (AOR = 1.73, 95% CI = 1.10–2.71, *p* = 0.027) were significantly associated with diarrhea. Contrary, children from socio-economically richer (AOR = 0.64, 95% CI = 0.45–0.91, *p* = 0.015) and household with unimproved water sources (AOR = 0.65, 95% CI = 0.46–0.91, *p* = 0.014) negatively impacted childhood diarrhea (Table 3).

For the outcome, fever, children aged 12–23 months (AOR = 1.90, 95% CI = 1.64–2.21, *p* = < 0.001), being underweight (AOR = 1.40, 95% CI = 1.24–1.55, *p* < 0.000) and fourth & above birth ordered children (AOR = 1.18, 95% CI = 1.01–1.38, *p* = 0.035) were associated with fever. On the other hand, the children of mothers with no formal education (when fathers were educated) had 30.0% (AOR = 0.70, 95% CI = 0.53–0.93, *p* = 0.015) lower chances of getting fever than children with educated parents (Table 3).

Similarly, children aged 12–23 months (AOR = 5.55, 95% CI = 3.15–9.77, *p* < 0.001); from the Rajshahi division (AOR = 2.29, 95% CI = 1.40–3.73, *p* = 0.001); being underweight (1.39, 95% CI = 1.01–1.91, *p* = 0.041) were associated with the coexistence of diarrhea and fever. On the other hand, children with educated mothers had 47.0% (AOR = 0.53, 95% CI= 0.30–0.92, *p* = 0.024) lower chances of having a coexistence of diarrhea and fever than children of educated parents (Table 3).

## 4. Discussion

The overall prevalence of diarrhea, fever, and coexistence of diarrhea and fever among children under-five were around 5.0%, 34.0%, and 3.0%, respectively in Bangladesh. The prevalence of diarrhea was higher in some neighboring countries, such as 29% in Afghanistan, 19% in Pakistan, and 10% in Myanmar, followed by 9% in India, 8% in Nepal, and 4% in Maldives [22,26]. Similarly, the prevalence of fever in Bangladesh was followed by Afghanistan (29%), Myanmar (24%) and Nepal (21%). However, the prevalence of fever was higher in Pakistan (38%) compared to Bangladesh [26]. While the reporting of the coexistence of diarrhea and fever was not usually common in Bangladesh, it is notable that the prevalence of the coexistence of multiple illnesses was higher in Pakistan compared to Bangladesh, regardless of the combination of diarrhea and fever [27,28]. In addition, the prevalence of diarrhea and coexistence of diarrhea and fever was significantly higher among children aged 12–23 months and fever was highly prevalent among children born with low birth weight.

This study also found that children less than 2 years of age, and mothers with no formal education were critical factors associated with childhood diarrhea. These factors were thoroughly examined and identified previously in Bangladesh, India, Pakistan, Nepal, and other African countries [9,27]. The physical and mental development of children under the age of two years are particularly sensitive [29,30,31,32]. Various factors such as inappropriate and imbalanced initiation of dietary patterns, an immature immune system, lack of previous exposure to pathogens like diarrheal-causing viruses, bacteria, or parasites, and a weakened digestive system in this age interval, all potentially have detrimental effects on their well-being. [29,30,31,32,33]. Despite notable advancements in women’s education at all levels in Bangladesh, a significant proportion of females in the country still face inadequate access to education [31]. Additionally, the combination of poor-quality education, limited coverage and implementation of health education, as well as poverty, can have an impact on children’s health [32,33]. The present study unveiled an intriguing and previously understated finding that children from the wealthiest socioeconomic backgrounds were found to be at a heightened risk of childhood diarrhea. This contradicts the prevailing notion in most studies, which primarily linked childhood illnesses with lower socioeconomic status [9,27]. Children from higher socioeconomic backgrounds, who may have better access to resources and living conditions, can still experience diarrhea due to various reasons, such as consumption of stored, processed, raw, or undercooked food, overuse or misuse of antibiotics, and imbalanced dietary patterns [34,35]. Furthermore, research findings indicated that children from the Barisal division (southern region) had a higher likelihood of experiencing diarrhea. This increased prevalence can be attributed to climate change, frequent natural disasters, salinity, and land degradation, which have limited dietary diversity and created a substantial burden of adverse health effects in this particular region [36]. Remarkably, it was identified that an improved source of drinking water was a significant contributing factor to diarrhea among children under-five in Bangladesh. This could be attributed to various reasons, such as contamination during storage and handling, insufficient sanitation practices, issues with piped water distribution, unsafe water treatment practices, and other related factors [37]. To mitigate the adverse health outcomes in children, it would be beneficial to design and implement effective health interventions, preventive measures, and coverage that incorporates evidence-based health education, awareness programs, maternal reproductive and child health education, as well as appropriate sanitation and hygiene practices.

Children less than 2 years of age, underweight children, fourth and above birth order children, educated parents, and moderate-to-high socio-economic status, and had significant effects on childhood fever. The findings partially concurred with the previous studies conducted in Bangladesh, India, and other African countries [5,9,17,21,38]. Childhood undernutrition was another key factor associated with increased illness [39]. Although there is limited research on the relationship between birth order and children’s health, studies have indicated that later-born children in larger families may face an elevated risk of hospitalization for infections, respiratory diseases, and issues related to the eyes and ears, possibly due to receiving less parental attention [40]. First-born children tend to receive more parental time and attention compared to their siblings, while subsequent children may receive less quality time during their early years. This discrepancy in parental attention can contribute to adverse health outcomes such as fever in children of higher birth order [41]. The educational attainment of both parents is often linked to a higher socioeconomic status, leading to both parents engaging in income-generating activities. This can result in limited availability of time for children and potentially poor parenting practices, which may have negative effects on children’s health [42].

The coexistence of diarrhea and fever and the factors associated with it in Bangladesh has not been well documented. The current study found that children less than two years of age, and underweight children were the protective factor against the coexistence of diarrhea and fever. In addition, only the mother’s educational status (the father had no formal education) rather than both parents’ educational status, and Rajshahi division (mid-western region) were the key factors associated with the coexistence of diarrhea and fever. Some of these results were consistent with previous studies irrespective of the outcome coexistence of diarrhea and fever [27,28]. The mid-western region (Rajshahi division) is one of the climate-vulnerable regions [43,44]. Frequent floods often record the highest temperature, contaminated groundwater, insufficient economic and social benefits, and poor access to basic needs pose a significant threat to human health resulting in poor child health outcomes [43,44,45]. Incorporating better management of climate disasters and water sources improved sanitation and hygiene, and educational intervention with poverty alleviation strategies might improve the situation of childhood illness in this region.

A remarkable finding in a recent study revealed that the educational background of both parents, their rich socioeconomic status, and improved toilet facilities were significant risk factors linked to childhood illness. This shift in the nature of risk factors, which now includes previously recognized protective factors, has created a challenging situation when addressing childhood illness. The lack of practical knowledge and awareness about illness, along with unhealthy lifestyles among household members, could be contributing to the relevant information gap. While existing policies heavily attached to tackling children’s adverse health outcomes in low resource setting, needs extensive review. Further research is necessary to explore deeper into this subject area.

The main strength of this study was the utilization of a nationally representative cross-sectional sample which covers both rural and urban areas of all districts of the country as well as aids in generalizing the findings in similar settings. Additionally, BDHS 2017–2018 data was collected by using a standard questionnaire, designing a complex survey strategy, and global study model to provide credible results. Despite these advantages, we acknowledged several limitations of this study. As the data was collected based on the mother’s self-reported retrospective information, the information might be affected by recall bias. The cross-sectional nature of the data interferes with drawing causal associations between dependent and independent variables.

## 5. Conclusions

In Bangladesh, the prevalence rates for diarrhea, fever, and the coexistence of these conditions among children can be expressed based on the findings: one in every twenty children experiences diarrhea, one in every three children experiences fever, and one out of every 35 children suffer from the coexistence of these conditions. Age of children, underweight children, birth order, parental education, wealth index, and region of residence were identified as key determinates of childhood illness. Both parents’ educational status and rich socio-economic status were remarkably identified as risk factors for childhood illness. Designing an evidence-based health intervention focusing on the current study findings, appropriate planning for the implementation, and incorporating a wide range of community participation from all social classes in both urban and rural areas can help in reducing childhood illness. The study also suggests extensive research and review of previous studies and policies.

## Figures and Tables

**Table 1:** Background characteristics of the study variables.

Variables	Number	(%)	No of Response/ Missing or Excluded
Total	7663	100.0	8759/1096
Age of the children			
0–11 months	1677	21.9	8759/447
12–23 months	1577	20.6	
24–35 months	1481	19.3	
36–47 months	1414	18.4	
48–59 months	1514	19.8	
Sex of the children			
Male	3996	52.2	8759/0
Female	3667	47.8	
Underweight children			
No	5972	77.9	8759/709
Yes	1691	22.1	
Birth order of the children			
First	2903	37.9	8759/0
Second	2507	32.7	
Third	1298	16.9	
Fourth and above	955	12.5	
Small birth weight ^#^			
No	1816	38.3	8759/3455
Yes	325	6.9	
Not weighted	2594	54.8	
Age of the mothers (in years)			
15–19	938	12.2	8759/0
20–24	2679	35.0	
25–29	2146	28.0	
30–34	1295	16.9	
34–39	481	6.3	
≥40	124	1.6	
Parents’ education			
Both parents uneducated	295	3.8	8759/153
Only father uneducated	865	11.3	
Only mother uneducated	243	3.2	
Both parents educated	6260	81.7	
Mother currently working			
No	4561	59.5	8759/0
Yes	3102	40.5	
Mothers received antenatal care ^#^			
No	364	8.0	8759/3747
Yes	4175	92.0	
Mothers received postnatal care ^#^			
No	1509	33.3	8759/3753
Yes	3025	66.7	
Mother’s decision-making autonomy			
No	6581	85.9	8759/154
Yes	1082	14.1	
Mass media exposure			
No	2774	36.2	8759/0
Yes	4889	63.8	
Source of drinking water			
Improved	6659	86.9	8759/0
Unimproved	1004	13.1	
Type of toilet facility			
Improved	4362	56.9	8759/0
Unimproved	3301	43.1	
Solid waste use in cooking			
No	2219	29.0	8759/6
Yes	5444	71.0	
Wealth index			
Poorest	1707	22.3	8759/0
Poorer	1545	20.2	
Middle	1382	18.0	
Richer	1535	20.0	
Richest	1494	19.5	
Place of residence			
Urban	2605	34.0	8759/0
Rural	5058	66.0	
Divisions in Bangladesh			
Barisal	800	10.4	8759/0
Chattogram	1246	16.2	
Dhaka	1079	14.1	
Khulna	810	10.6	
Mymensingh	911	11.9	
Rajshahi	796	10.4	
Rangpur	879	11.5	
Sylhet	1142	14.9	

^#^, information available only for children aged 0–23 months.

**Table 2:** Prevalence of under-5 child acute morbidity and co-morbidity.

Variables	Diarrhea		Fever		Coexistence of Diarrhes and Fever	
	*n*	Prevalence (95% CI)	*n*	Prevalence (95% CI)	*n*	Prevalence (95% CI)
Total	386	4.9 (4.3–5.5)	2561	33.7 (32.3–35.2)	218	2.7 (2.3–3.2)
Age of the children						
0–11 months	101	5.7 (4.5–7.2)	608	36.8 (34.3–39.5)	63	3.5 (2.6–4.7)
12–23 months	147	9.1 (7.7–10.8)	635	40.0 (37.0–43.1)	77	4.8 (3.8–6.1)
24–35 months	73	5.0 (3.9–6.3)	488	33.3 (30.5–36.3)	41	2.8 (2.0–3.8)
36–47 months	38	2.5 (1.8–3.5)	413	29.4 (26.8–32.1)	20	1.2 (0.8–2.0)
48–59 months	27	1.5 (1.0–2.3)	417	27.9 (25.2–30.7)	17	1.0 (0.6–1.7)
Chi-square *p* values	*p* < 0.001		*p* < 0.001		*p* < 0.001	
Sex of the children						
Male	221	5.3 (4.5–6.2)	1357	34.4 (32.7–36.3)	123	3.0 (2.5–3.7)
Female	165	4.4 (3.7–5.2)	1204	32.9 (30.9–34.9)	95	2.4 (1.9–3.0)
Chi-square *p* values	*p* = 0.116		*p* = 0.200		*p* = 0.130	
Underweight children						
No	304	4.8 (4.2–5.5)	1908	32.4 (30.8–34.0)	163	2.6 (2.2–3.1)
Yes	82	5.1 (4.0–6.5)	653	38.5 (35.8–41.3)	55	3.3 (2.4–4.4)
Chi-square *p* values	*p* = 0.678		*p* = 0.0001		*p* = 0.170	
Birth order of the children						
First	143	4.8 (4.0–5.7)	909	31.8 (29.7–33.9)	78	2.5 (2.0–3.2)
Second	144	5.6 (4.7–6.8)	850	33.9 (31.7–36.2)	87	3.4 (2.6–4.3)
Third	63	4.8 (3.7–6.1)	454	35.2 (32.4–38.1)	36	2.9 (2.0–4.0)
Fourth and above	36	3.4 (2.3–4.9)	348	37.0 (33.4–40.7)	17	1.6 (0.9–2.8)
Chi-square *p* values	*p* = 0.088		*p* = 0.036		*p* = 0.057	
Small birth weight						
No	134	6.9 (5.7–8.3)	637	35.1 (32.7,37.6)	77	4.0 (3.1–5.0)
Yes	26	7.7 (5.1–11.5)	132	41.8 (35.6–48.2)	12	4.0 (2.1–7.2)
Not weighted	163	6.3 (5.4–7.4)	964	37.4 (35.2–39.6)	93	3.5 (2.8–4.4])
Chi-square *p* values	*p* = 0.616		*p* = 0.087		*p* = 0.776	
Age of the mothers (in years)						
15–19	61	6.4 (4.9–8.3)	352	37.1 (33.5–40.9)	39	3.9 (2.8–5.5)
20–24	141	5.2 (4.3–6.2)	857	32.5 (30.4–34.7)	69	2.6 (2.0–3.3)
25–29	120	5.3 (4.3–6.4)	732	33.8 (31.4–36.3)	66	2.8 (2.1–3.6)
30–34	46	3.3 (2.4–4.6)	428	33.7 (30.8–36.7)	32	2.5 (1.6–3.7)
34–39	12	2.7 (1.5–4.8)	153	33.7 (29.0–38.6)	6	1.3 (0.5–3.0)
≥40	6	3.8 (1.5–8.9)	39	30.4 (22.2–40.1)	6	3.8 (1.5–8.9)
Chi-square *p* values	*p* = 0.008		*p* = 0.301		*p* = 0.114	
Parents’ education						
Both parents uneducated	10	4.8 (2.5–8.9)	105	34.4 (28.5–40.7)	6	2.8 (1.2–6.2)
Only father uneducated	34	3.5 (2.4–4.9)	276	32.4 (28.9–36.0)	16	1.5 (0.9–2.6)
Only mother uneducated	20	8.7 (5.5–13.4)	73	29.3 (23.5–35.8)	10	3.7 (1.9,7.2)
Both parents educated	322	4.9 (4.3–5.6)	2107	34.0 (32.4–35.7)	186	2.9 (2.4–3.4)
Chi-square *p* values	*p* = 0.032		*p* = 0.467		*p* = 0.172	
Mother currently working						
No	242	5.1 (4.5–5.9)	1552	34.4 (32.6–36.2)	139	3.0 (2.5–3.5)
Yes	144	4.5 (3.7–5.4)	1009	32.7 (30.7–34.8)	79	2.4 (1.9–3.1)
Chi-square *p* values	*p* = 0.246		*p* = 0.214		*p* = 0.175	
Mothers received antenatal care						
No	27	7.5 (4.9–11.3)	138	39.0 (32.8–45.4)	13	3.3 (1.8–5.8)
Yes	285	6.6 (5.8–7.5)	1533	36.8 (35.0–38.7)	163	3.8 (3.2–4.5)
Chi-square *p* values	*p* = 0.216		*p* = 0.518		*p* = 0.623	
Mothers received postnatal care						
No	100	6.8 (5.6–8.4)	530	34.7 (32.1–37.5)	55	3.7 (2.8–4.8)
Yes	212	6.6 (5.7–7.7)	1140	38.2 (36.0–40.4)	121	3.8 (3.1–4.7)
Chi-square *p* values	*p* = 0.819		*p* = 0.040		*p* = 0.822	
Mother’s decision-making autonomy						
No	51	4.6 (3.4–6.2)	379	34.7 (31.3–38.4)	192	2.8 (2.3–3.2)
Yes	335	4.9 (4.3–5.6)	2182	33.5 (32.0–35.1)	26	2.5 (1.7–3.8)
Chi-square *p* values	*p* = 0.091		*p* = 0.009		*p* = 0.255	
Mass media exposure						
No	124	4.5 (3.6–5.6)	952	34.2 (32.0–36.5)	68	2.6 (1.9–3.5)
Yes	262	5.1 (4.4–5.8)	1609	33.4 (31.7–35.2)	150	2.8 (2.3–3.4)
Chi-square *p* values	*p* = 0.395		*p* = 0.545		*p* = 0.664	
Source of water						
Improved	351	5.0 (4.5–5.7)	2247	34.0 (32.5–35.5)	195	2.8 (2.4–3.3)
Unimproved	35	3.7 (2.7–5.3)	314	31.6 (28.4–35.0)	23	2.4 (1.5–3.7)
Chi-square *p* values	*p* = 0.089		*p* = 0.180		*p* = 0.486	
Type of toilet facility						
Improved	228	5.0 (4.3–5.8)	1450	33.7 (31.9–35.5)	129	2.8 (2.3–3.4)
Unimproved	158	4.7 (3.9–5.6)	1111	33.7 (31.8–35.7)	89	2.6 (2.0–3.3)
Chi-square *p* values	*p* = 0.522		*p* = 0.952		*p* = 0.623	
Solid waste used in cooking						
No	99	4.2 (3.4–5.2)	669	31.1 (28.8–33.6)	56	2.4 (1.8–3.2)
Yes	287	5.2 (4.5–5.9)	1892	34.8 (33.1–36.5)	162	2.9 (2.4–3.4)
Chi-square *p* values	*p* = 0.091		*p* = 0.009		*p* = 0.255	
Wealth index						
Poorest	84	4.9 (3.8–6.2)	600	35.0 (32.5–37.5)	47	2.7 (2.0–3.8)
Poorer	77	4.8 (3.8–6.1)	522	33.5 (30.7–36.5)	42	2.7 (2.0–3.7)
Middle	84	6.1 (4.7–7.9)	471	35.0 (32.0–38.1)	45	3.4 (2.4–4.6)
Richer	58	3.4 (2.6–4.5)	537	35.0 (32.1–38.0)	40	2.2 (1.5–3.1)
Richest	83	5.3 (4.1–6.7)	431	29.6 (26.8–32.6)	44	2.7 (2.0–3.7)
Chi-square *p* values	*p* = 044		*p* = 0.034		*p* = 0.484	
Place of residence						
Urban	132	4.6 (3.7–5.7)	805	31.8 (29.4–34.3)	72	2.7 (2.0–3.6)
Rural	254	5.0 (4.3–5.7)	1756	34.4 (32.6–36.2)	146	2.8 (2.3–3.3)
Chi-square *p* values	*p* = 0.567		*p* = 0.095		*p* = 0.833	
Divisions in Bangladesh						
Barisal	51	6.5 (5.0–8.3)	296	38.3 (34.0–42.7)	24	3.1 (2.0–4.7)
Chattogram	67	5.3 (4.1–7.0)	412	33.3 (30.0–36.7)	43	3.3 (2.3–4.7)
Dhaka	44	4.1 (3.0–5.4)	340	31.8 (28.5–35.2)	19	1.7 (1.1–2.7)
Khulna	33	3.9 (2.8–5.4)	238	31.2 (27.1–35.6)	18	2.2 (1.4–3.5)
Mymensingh	47	5.0 (3.7–6.7)	301	33.7 (29.9–37.7)	30	3.2 (2.2–4.5)
Rajshahi	49	6.1 (4.6–8.1)	278	35.5 (30.9–40.3)	29	3.6 (2.4–5.4)
Rangpur	37	4.5 (3.0–6.7)	312	36.4 (32.2–40.7)	22	2.7 (1.6–4.4)
Sylhet	58	4.8 (3.5–6.6)	384	34.3 (31.4–37.2)	33	2.9 (2.0–4.2)
Chi-square *p* values	*p* = 0.239		*p* = 0.297		*p* = 0.124	

Notes: CI, Confidence Interval; *n*, number of children.

**Table 3:** Risk factors of under-five child diarrhea, fever, and coexistence of diarrhea and fever.

Risk Factors	Diarrhea		Fever		Coexistence of Diarrhea and Fever	
	AOR (95% CI)	*p* Values	AOR (95% CI)	*p* Values	AOR (95% CI)	*p* Values
Age of the children						
0–11 months	4.07 (2.57–6.43)	<0.001	1.58 (1.36–1.84)	<0.001	4.05 (2.27–7.22)	<0.001
12–23 months	6.78 (4.35–10.54)	<0.001	1.90 (1.64–2.21)	<0.001	5.55 (3.15–9.77)	<0.001
24–35 months	3.61 (2.27–5.74)	<0.001	1.34 (1.15–1.56)	<0.001	3.00 (1.65–5.44)	<0.001
36–47 months	1.71 (1.02–2.88)	0.041	1.14 (0.98–1.34)	0.082	1.38 (0.70–2.74)	0.347
48–59 months^®^	1.00		1.00		1.00	
Underweight children						
No^®^			1.00		1.00	
Yes			1.40 (1.24–1.55)	<0.001	1.39 (1.01–1.91)	0.041
Birth order of the children						
First^®^			1.00			
Second			1.09 (0.97–1.22)	0.117		
Third			1.16 (1.01–1.33)	0.031		
Fourth and above			1.18 (1.01–1.38)	0.035		
Parents’ education						
Both parents uneducated	1.08 (0.63–1.88)	0.759	0.83 (0.64–1.07)	0.166	0.99 (0.48–2.03)	0.980
Only father uneducated	0.67 (0.46–0.99)	0.049	0.86 (0.74–1.00)	0.062	0.53 (0.30–0.92)	0.024
Only mother uneducated	1.94 (1.23–3.07)	0.004	0.70 (0.53–0.93)	0.015	1.60 (0.85–2.99)	0.141
Both parents educated	1.00		1.00		1.00	
Source of drinking water						
Improved^®^	1.00					
Unimproved	0.65 (0.46–0.91)	0.014				
Wealth index						
Poorest	0.95 (0.67–1.34)	0.781	1.30 (1.12–1.52)	0.001		
Poorer	0.90 (0.641.27)	0.583	1.23 (1.05–1.43)	0.008		
Middle	1.18 (0.85–1.62)	0.305	1.32 (1.32–1.54)	<0.001		
Richer	0.64 (0.45–0.91)	0.015	1.54 (1.17–1.17)	<0.001		
Richest^®^	1.00		1.00			
Divisions in Bangladesh						
Dhaka^®^	1.00				1.00	
Barisal	1.73 (1.10–2.71)	0.017			2.08 (1.12–3.86)	0.020
Chattogram	1.32 (0.97–1.81)	0.075			1.97 (1.28–3.05)	0.020
Khulna	1.07 (0.69–1.66)	0.746			1.48 (0.82–2.66)	0.186
Mymensingh	1.33 (0.87–2.02)	0.176			1.97 (1.14–3.42)	0.015
Rajshahi	1.61 (1.12–2.32)	0.009			2.29 (1.40–3.73)	0.001
Rangpur	1.13 (0.75–1.71)	0.535			1.73 (1.73–2.95)	0.041
Sylhet	1.25 (0.81–1.92)	0.309			1.75 (0.98–3.11)	0.055

Notes: AOR, Adjusted odds ratio; CI, Confidence Interval and ^®^, Reference Category.

## Data Availability

The BDHS 2017–2018 data is publicly available on the DHS Program’s page at https://dhsprogram.com/data/ (accessed on 29 November 2021).

## Supplementary Materials

The following supporting information can be downloaded at: https://www.mdpi.com/article/10.3390/children10111829/s1, Table S1: Measurements of independent variables.

## References

[1] Mahumud R.A., Alam K., Renzaho A.M., Sarker A.R., Sultana M., Sheikh N., Rawal L.B., Gow J. (2019). Changes in inequality of childhood morbidity in Bangladesh 1993–2014: A decomposition analysis. PLoS ONE.

[2] WHO (2017). Diarrhoeal Disease.

[3] MacGill M. (2020). What You Should Know about Diarrhea. https://www.medicalnewstoday.com/articles/158634.php.

[4] UNICEF (2023). Under-Five Mortality.

[5] Antillón M., Warren J.L., Crawford F.W., Weinberger D.M., Kürüm E., Pak G.D., Marks F., Pitzer V.E. (2017). The burden of typhoid fever in low-and middle-income countries: A meta-regression approach. PLoS Neglected Trop. Dis..

[6] Ogoina D. (2011). Fever, fever patterns and diseases called ‘fever’—A review. J. Infect. Public Health.

[7] NIPORT (2020). Bangladesh Demographic and Health Survey 2017–18.

[8] Pinzón-Rondón Á.M., Zárate-Ardila C., Hoyos-Martínez A., Ruiz-Sternberg Á.M., Vélez-van-Meerbeke A. (2015). Country characteristics and acute diarrhea in children from developing nations: A multilevel study. BMC Public Health.

[9] Rahman A., Hossain M.M. (2022). Prevalence and determinants of fever, ARI and diarrhea among children aged 6–59 months in Bangladesh. BMC Pediatr..

[10] MoHFW (2015). Success Factors for Women’s and Children’s Health: Bangladesh.

[11] WB (2021). Mortality Rate, under-5 (per 1000 Live Births).

[12] BBS (2016). Report on Sample Vital Registration System-2015.

[13] Thiam S., Diène A.N., Fuhrimann S., Winkler M.S., Sy I., Ndione J.A., Schindler C., Vounatsou P., Utzinger J., Faye O. (2017). Prevalence of diarrhoea and risk factors among children under five years old in Mbour, Senegal: A cross-sectional study. Infect. Dis. Poverty.

[14] Woldu W., Bitew B.D., Gizaw Z. (2016). Socioeconomic factors associated with diarrheal diseases among under-five children of the nomadic population in northeast Ethiopia. Trop. Med. Health.

[15] Amugsi D.A., Aborigo R.A., Oduro A.R., Asoala V., Awine T., Amenga-Etego L. (2015). Socio-demographic and environmental determinants of infectious disease morbidity in children under 5 years in Ghana. Glob. Health Action.

[16] Solomon E.T., Gari S.R., Kloos H., Mengistie B. (2020). Diarrheal morbidity and predisposing factors among children under 5 years of age in rural East Ethiopia. Trop. Med. Health.

[17] Kamal M.M., Hasan M.M., Davey R. (2015). Determinants of childhood morbidity in Bangladesh: Evidence from the demographic and health survey 2011. BMJ Open.

[18] Schlaudecker E.P., Steinhoff M.C., Moore S.R. (2011). Interactions of diarrhea, pneumonia, and malnutrition in childhood: Recent evidence from developing countries. Curr. Opin. Infect. Dis..

[19] Banga D., Baren M., Ssonko N.V., Sikakulya F.K., Tibamwenda Y., Banga C., Ssebuufu R. (2020). Comorbidities and factors associated with mortality among children under five years admitted with severe acute malnutrition in the nutritional unit of Jinja Regional Referral Hospital, Eastern Uganda. Int. J. Pediatr..

[20] WHO (2019). More Women and Children Survive Today than ever before—UN Report.

[21] Adedokun S.T., Yaya S. (2020). Childhood morbidity and its determinants: Evidence from 31 countries in sub-Saharan Africa. BMJ Glob. Health.

[22] Paul P. (2020). Socio-demographic and environmental factors associated with diarrhoeal disease among children under five in India. BMC Public Health.

[23] Bursac Z., Gauss C.H., Williams D.K., Hosmer D.W. (2008). Purposeful selection of variables in logistic regression. Source Code Biol. Med..

[24] Thiese M.S., Ronna B., Ott U. (2016). P value interpretations and considerations. J. Thorac. Dis..

[25] Kim J. (2015). How to Choose the Level of Significance: A Pedagogical Note.

[26] DHS (2015). Children with Fever and Diarrhoea. The Demographic and Health Surveys (DHS) Program STATcompiler ICF. Funded by USAID. http://www.statcompiler.com.

[27] Hasan M.M., Richardson A. (2017). How sustainable household environment and knowledge of healthy practices relate to childhood morbidity in South Asia: Analysis of survey data from Bangladesh, Nepal and Pakistan. BMJ Open.

[28] Rashmi R., Paul R. (2022). Determinants of multimorbidity of infectious diseases among under-five children in Bangladesh: Role of community context. BMC Pediatr..

[29] Chowdhury M.R.K., Rahman M.S., Khan M.M.H. (2016). Levels and determinants of complementary feeding based on meal frequency among children of 6 to 23 months in Bangladesh. BMC Public Health.

[30] URMC (2023). Viruses, Bacteria, and Parasites in the Digestive Tract. Health Encyclopedia.

[31] Al-Zayed S.R., Talukdar F., Jahan F., Asaduzzaman T., Shams F. (2018). Beyond Gender Parity: Actualization of Benefits Verses Fallacy of Promises, a Case Study of Bangladesh.

[32] Zaidman E.A., Scott K.M., Hahn D., Bennett P., Caldwell P.H. (2023). Impact of parental health literacy on the health outcomes of children with chronic disease globally: A systematic review. J. Paediatr. Child Health.

[33] Houweling T.A., Kunst A.E., Looman C.W., Mackenbach J.P. (2005). Determinants of under-5 mortality among the poor and the rich: A cross-national analysis of 43 developing countries. Int. J. Epidemiol..

[34] Signs R.J., Darcey V.L., Carney T.A., Evans A.A., Quinlan J.J. (2011). Retail food safety risks for populations of different races, ethnicities, and income levels. J. Food Prot..

[35] Newman K., Leon J., Rebolledo P., Scallan E. (2015). The impact of socioeconomic status on foodborne illness in high-income countries: A systematic review. Epidemiol. Infect..

[36] Kabir R., Khan H.T., Ball E., Caldwell K. (2016). Climate change impact: The experience of the coastal areas of Bangladesh affected by cyclones Sidr and Aila. J. Environ. Public Health.

[37] Hutton G., Chase C. (2017). Water supply, sanitation, and hygiene. Injury Prevention and Environmental Health.

[38] John J., Bavdekar A., Rongsen-Chandola T., Dutta S., Kang G. (2018). Estimating the incidence of enteric fever in children in India: A multi-site, active fever surveillance of pediatric cohorts. BMC Public Health.

[39] Walson J.L., Berkley J.A. (2018). The impact of malnutrition on childhood infections. Curr. Opin. Infect. Dis..

[40] Björkegren E., Svaleryd H. (2017). Birth Order and Child Health.

[41] Price J. (2008). Parent-child quality time: Does birth order matter?. J. Hum. Resour..

[42] Friedman S.D. (2018). How Our Careers Affect Our Children. Harvard Business Review.

[43] Haque A.M., Salehin M.A. (2019). Climate Change in Bangladesh: Effect versus Awareness of the Local People and Agencies of Rajshahi City. Soc. Sci. J..

[44] CDKN Global Urban Development Must Be Planned and Climate-Resilient—Experience from Rajshahi City, Bangladesh. Climate and Development Knowledge Network (CDKN) Global, Bangladesh 2020. https://cdkn.org/story/feature-urban-development-must-be-planned-and-climate-resilient-experience-from-rajshahi-city-bangladesh.

[45] Alam M.Z., Rahman M.A., Al Firoz M.A. (2013). Water supply and sanitation facilities in urban slums: A case study of Rajshahi City corporation slums. Am. J. Civ. Eng. Archit..

