# Supplementary material for: Prevalence and Determinants of Diarrhea, Fever, and Coexistence of Diarrhea and Fever in Children Under-Five in Bangladesh
Source: Children (Basel). 2023 Nov 20;10(11):1829. doi: 10.3390/children10111829 (PMC10670412; doi:10.3390/children10111829)
Supplement: Supplementary file 1 [file children-10-01829-s001.zip › children-2695224-supplementary.pdf]

Supplementary Table

Table S1. Measurements of independent variables

Variable	Description	Measurement	Scale of measurement
Mothers' age (in years)	Age of mothers at the time of data collection	15-19, 20-24, 25-29, 30-34, 35-39, ≥ 40	Discrete, categorial
Parents' education	Parental educational status can be categorized as "educated" when they have completed a minimum of five years of schooling, typically corresponding to the primary level (class 1 to 5). On the other hand, if there are no schooling years completed (0 years of schooling), this categorizes them as "uneducated."	Both parents were uneducated, only father was uneducated, only mother was uneducated, both parents were educated	Categorical
Mother's currently working	Mother engaged in economic activity at the time of data collection	Not working, Currently working	Binary
Mothers received antenatal care	At least one medical surveillance and review performed during pregnancy for the early detection of possible complications of pregnancy	No, Yes	Binary
Mothers received postnatal care	At least one postnatal care is the individualized care provided to meet the needs of a mother and her baby following childbirth	No, Yes	Binary
Mothers' decision-making autonomy	In the BDHS surveys, a woman's decision-making power is assessed through three key aspects: 1) a woman who usually decides on her healthcare 2) a woman who usually decides on large household purchases and 3) a woman who usually decides on visits to family or relatives. The response options were as follows: (a) respondent alone, (b) respondent and husband/partner, (c) respondent and another person, (d) husband/partner alone, (e) someone else, (f) other. For each question, a score of 1 is assigned to indicate an inability to make decisions when the responses are "d," "e," or "f," and a score of 0 is given otherwise when the responses are "a," "b," or "c." These scores are then aggregated, resulting in a cumulative score ranging from 0 to 3. The high internal consistency of the instruments is reflected in the Cronbach's α value of 0.79, indicating strong reliability in the measurement of decision-making power. Further, 0 was defined ability to participate in decision making while 1-3 indicated not able to participate.	Not participated, Participated	Binary
Children's age (in months)	Age of the children at the time of data collection	0-11 months, 12-23 months, 24-35 months, 36-47 months, 48-59 months	Categorical
Sex of child	Sex differential of children	Male, Female	Binary
Birth order	Birth order is the chronological order of sibling's birth in a family	One, two, three, four and above	Categorical

Small birth weight	Children were <2.5 kg of weight during birth.	No, Yes, Not weighted	Categorical
Mass media exposure	Mass media exposure including television, radio, and newspapers/magazines, is characterized as regular exposure to any of these media outlets at least once a week.	No, Yes	Binary
Source of water	Improved water sources include piped water supplied directly to the dwelling, piped water to a yard or plot, access to a public tap or standpipe, piped water to a neighbouring source, tube wells or boreholes, protected wells, protected springs, rainwater collection, tanker truck delivery, carts with small water tanks, and bottled water.<br>Unimproved water sources encompass unprotected wells, unprotected springs, and surface water from sources such as rivers, dams, lakes, ponds, streams, canals, and irrigation channels, along with other non-protected sources.	Improved, unimproved	Binary
Type of toilet facility	Improved: flush - to piped sewer system, flush - to septic tank, flush - to pit latrine, flush - don't know where, pit latrine - ventilated improved pit, pit latrine - with slab, composting toilet; unimproved: flush - to somewhere else, pit latrine - without slab / open pit, bucket toilet, hanging toilet/latrine, other	Improved, unimproved	Binary
Solid waste use in cooking	Improved sanitation facilities include flush toilets connected to a piped sewer system, flush toilets linked to a septic tank, flush toilets connected to a pit latrine, flush toilets where the destination is unknown, ventilated improved pit latrines, pit latrines with a slab, and composting toilets.<br>Unimproved sanitation facilities encompass flush toilets leading to an unspecified location, pit latrines without a slab or in the form of open pits, bucket toilets, hanging toilets/latrines, and other non-standard facilities.	Clean fuel, Solid fuel	Binary
Wealth index	In DHS surveys, the wealth index is determined by the DHS authority through a process that involves household attributes and asset data, employing principal component analysis. Subsequently, households are categorized into quintiles based on their respective wealth index scores, with those having lower index values classified as the poorest, and conversely, those with higher values as the wealthiest.	Poorest, poorer, middle, richer, richest	Categorical
Place of residence	Different residential areas across the country.	Urban, rural	Binary

Division	Administrative regions of Bangladesh	Barisal, Chattogram, Dhaka, Khulna, Mymensingh, Rajshahi, Rangpur, Sylhet	Categorical
